# MiR-29b inhibits the growth of glioma via MYCN dependent way

**DOI:** 10.18632/oncotarget.16780

**Published:** 2017-04-01

**Authors:** Guan Sun, Jingmin Lu, Chuang Zhang, Ran You, Lei Shi, Nan Jiang, Dekang Nie, Jian Zhu, Min Li, Jun Guo

**Affiliations:** ^1^ Department of Neurosurgery, First People's Hospital of Yancheng, Fourth Affiliated Hospital of Nantong University, Yancheng 224001, PR China; ^2^ Department of Neurology, The Affiliated Huai'an Hospital of Xuzhou Medical University and The Second People's Hospital of Huai'an, Huai'an 223001, PR China; ^3^ Department of Medical Oncology, The 81st Hospital of People's Liberation Army, Nanjing 210002, PR China; ^4^ Department of Interventional Radiology, Nantong University Affiliated Hospital, Nantong 226000, PR China; ^5^ Department of Neurosurgery, The First People's Hospital of Kunshan Affiliated with Jiangsu University, Suzhou 215300, PR China; ^6^ Department of Neurosurgery, Jiangning Hospital Affiliated with Nanjing Medical University, Nanjing 211100, PR China

**Keywords:** glioma, miR-29b, MYCN

## Abstract

MiR-29b is widely involved in diverse cancers. We plan to study its role in glioma. The expression of miR-29b was detected by real-time polymerase chain reaction (PCR) and we found the expression of miR-29b was decreased in glioma. Cell proliferation was evaluated by cell counting kit (CCK8) and 5-Ethynyl-2′- deoxyuridine (EdU) and cell apoptosis was assayed with flow cytometry assay (FCA), which indicated miR-29b can inhibit the proliferation and promote the apoptosis of glioma cells. The target of miR-29b was predicted using miRanda, TargetScan and PicTar sofeware and we also found MYCN was a direct target of miR-29b in glioma cells and miR-29b inhibited the proliferation of glioma cells via MYCN dependent way. Subcutaneous xenotransplantation model was designed to investigate the affection of miR-29b on glioma growth. The effectiveness of miR-29b for glioma prediction was also performed and we determined miR-29b can stably exist and may act as a biomarker for the diagnosis of glioma. As a conclusion, miR-29b inhibits the growth of glioma via MYCN dependent way and can be a biomarker for the diagnosis of glioma.

## INTRODUCTION

Glioma, as one of the most common malignant tumors in human adults, accounts for 80% of all malignant brain tumors [1, 2]. In clinical, glioma is generally classified into four different grades (grade I to grade IV) according to the World Health Organization (WHO) grading system. Glioma diagnosed as high-grade (grade IV) is also known as glioblastoma multiforme (GBM), which is characterized by easy recurrence and unfavorable median overall survival of ∼15 months [3, 4]. Modern therapeutic approaches, including surgical resection, chemotherapy and radiotherapy have been developed and widely utilized; however, the prognosis of glioma patients has not been significantly improved [5–7].

The biological molecular therapies developing recently have provided promising strategies for the treatment of glioma, especially for high-grade glioma which cannot be cured through surgical resection [6, 8]. As candidate therapeutic targets, miRNAs are generally involved in various physical and pathological processes, including cell proliferation, apoptosis and differentiation and the pivotal roles of miRNAs in the development and progression of tumors are also widely studied.

The miR-29 family is consisted of three mature members, miR-29a, miR-29b and miR-29c, which are aberrantly down-regulated in several tumors [9]. Several studies have reported that miR-29b was high expressed in GBM cell lines and in serum of GBM patients, thus can participate in GBM and act as a biomarker for GBM diagnosis; however the precise mechanism of miR-29b in fine tuning the occurrence and progression of glioma has not been well illuminated [10, 11].

Bioinformatics analyses are widely used to investigate miRNAs within the human genome and identify putative candidate genes involved in carcinogenesis. Based on such methods, we found an important gene involved in glioma, MYCN, could be a key target of miR-29b in the development and progression of glioma. In this study, we further validated the decreased level of miR-29b in glioma tissues and demonstrated its functional roles in inhibiting the growth of glioma via MYCN dependent way.

## RESULTS

### Expression of miR-29b was decreased in glioma

**Figure 1:**
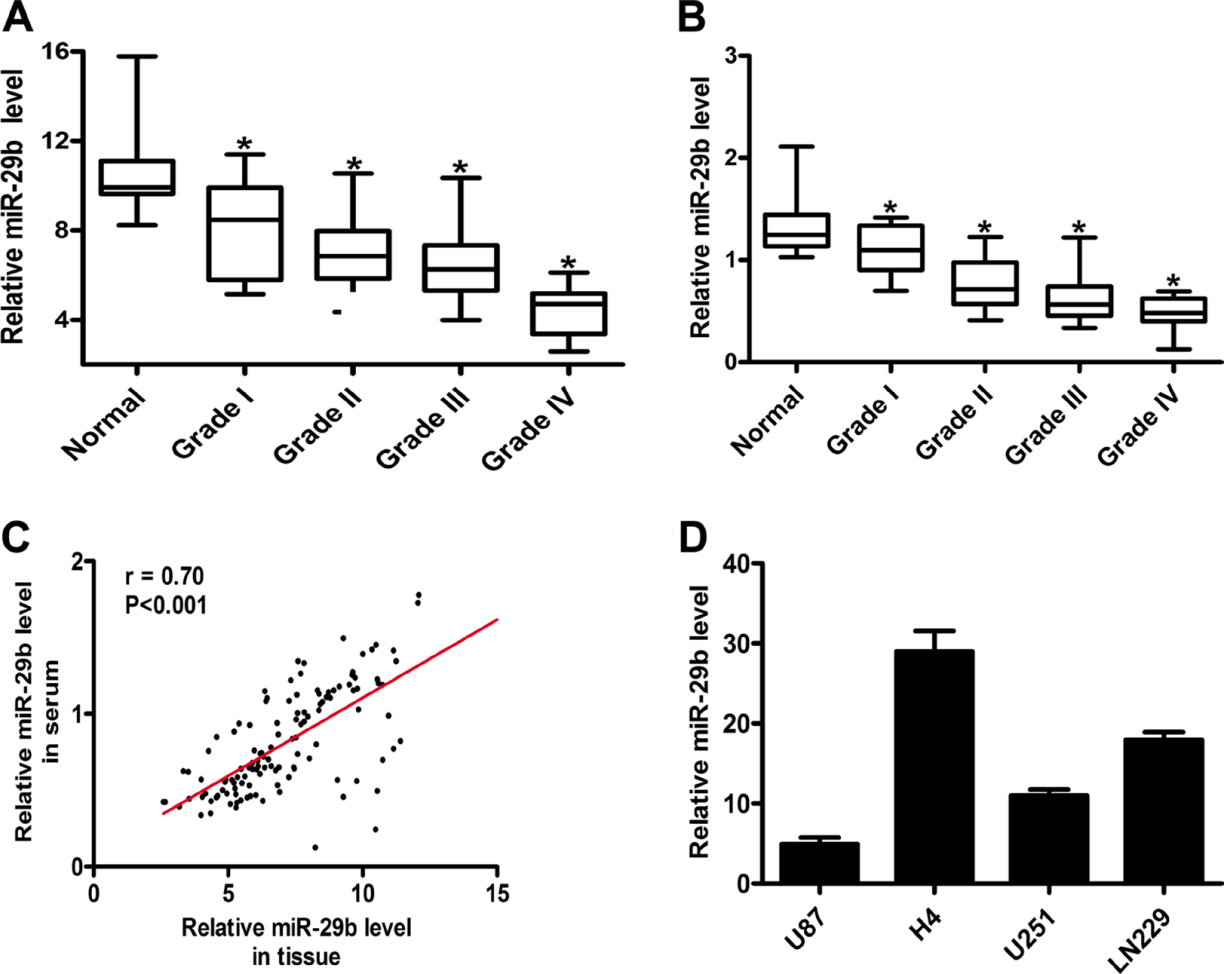
MiR-29b was down-regulated in glioma, detected by real-time PCR (**A**) Relative miR-29b level in normal brain (*n* = 20) and glioma tissues (*n* = 104). (**B**) Relative miR-29b level in glioma (*n* = 104) and healthy (*n* = 20) serum samples. Data were presented as box plots. Box plot explanation: upper horizontal line of box, 75th percentile; lower horizontal line of box, 25th percentile; horizontal bar within box, median; upper horizontal bar outside box, 95th percentile; lower horizontal bar outside box, 5th percentile. (**C**) The correlation of miR-29b levels in glioma tissues and corresponding serum samples. (**D**) Relative miR-29b level in glioma cell lines. Data were presented as the mean ± S.E.M. U6 was used as internal control. Data were based on at least three independent experiments. *P < 0.05 vs. control group.

We collected 20 normal brain tissues and 104 glioma tissues, including 14 samples in grade I, 48 samples in grade II, 26 samples in grade III and 16 samples in grade IV. The expression levels of miR-29b were detected by quantitative real-time PCR, and the results showed that in normal brain tissues the expression of miR-29b exhibited observably high level, while in glioma samples (WHO I, II, III and IV), the levels of miR-29b were significantly down-regulated. (Figure 1A) The expression levels of miR-29b in plasma were also evaluated, which exhibited the similar results in accordance with those in tissues. (Figure 1B) The correlation analysis was estimated, and we found that the expression levels miR-29b showed significant relevance in glioma tissues and in corresponding plasma samples. (Figure 1C) These results consistently indicated that the abnormal down-regulation of miR-29b commonly existed in glioma.

### MiR-29b inhibits the proliferation and promotes the apoptosis of glioma cells

**Figure 2:**
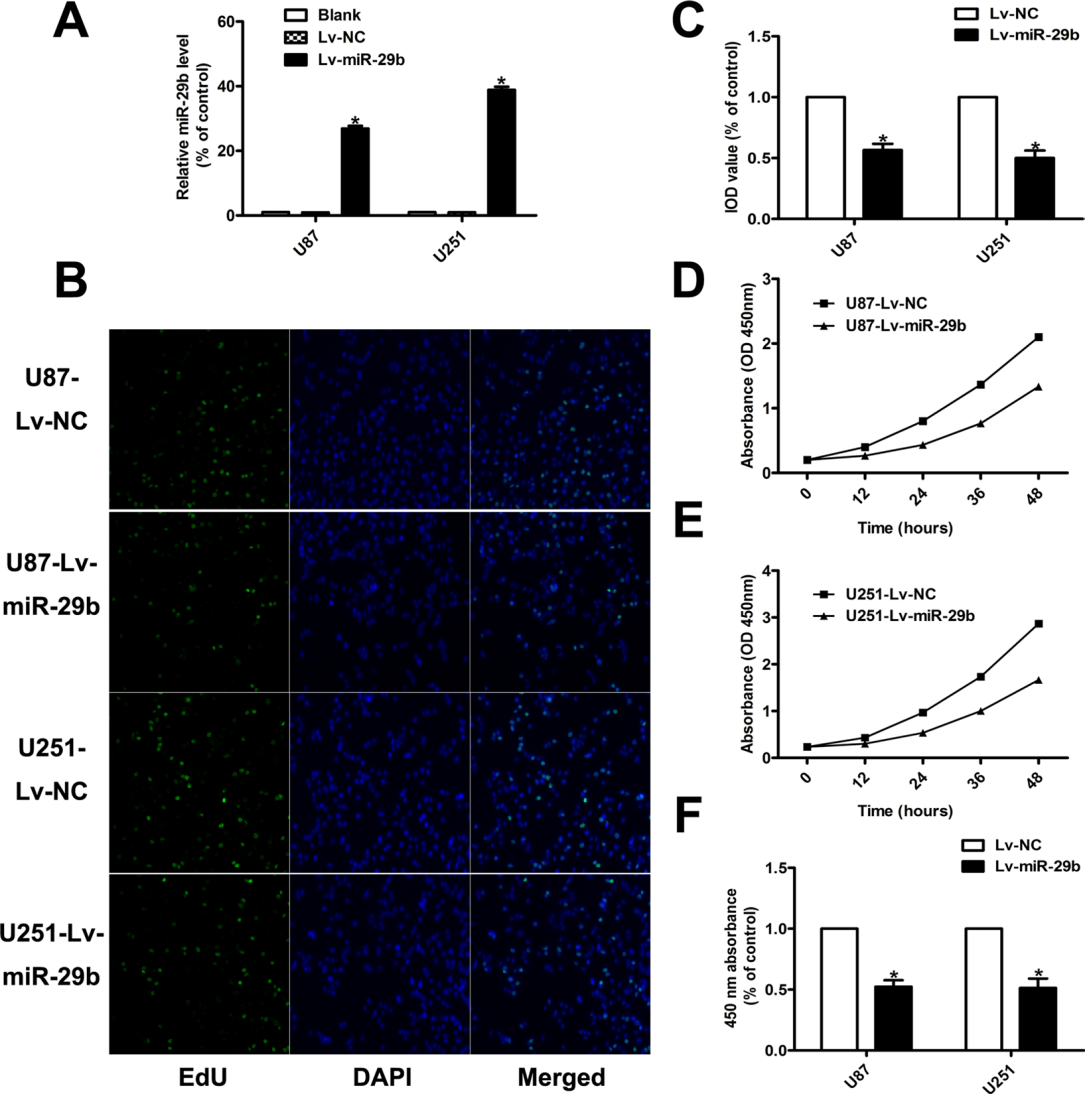
MiR-29b inhibited the proliferation ability of glioma cells (**A**) Validation results of real-time PCR for miR-29b over-expression. U6 was used as internal control. (**B**, **C**) EdU results indicated over-expression of miR-29b inhibited cell proliferation (*n* = 4). Data were presented as mean ± S.E.M. (**D**–**F**) CCK8 results demonstrated miR-29b over-expression inhibited cell proliferation. Absorbance at 450 nm was presented as the mean ± SEM. Absorbance at 450 nm of cells treated with control plasmid was normalized to 100%. Data were collected and provided at 24 h after cultivation. (*n* = 4) Data were based on at least three independent experiments. *P < 0.05 vs. control group.

**Figure 3:**
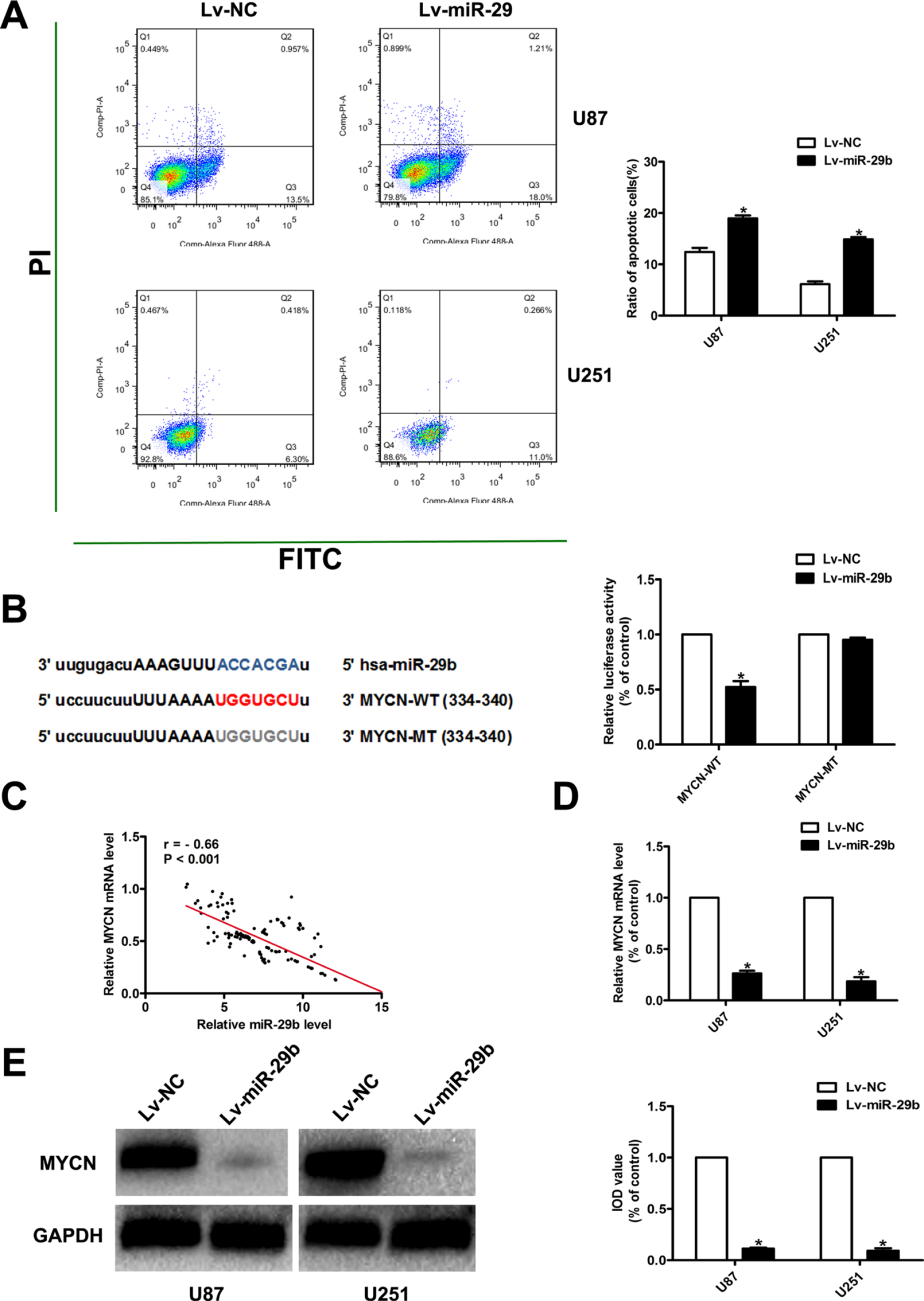
MiR-29b promoted the apoptosis of glioma cells; MiR-29b target prediction (**A**) Cell apoptosis was assayed by FCA. Left panel: PI for cell nucleus staining; FITC for cytomembrane staining. Right panel: Ratio of early-phase apoptotic cells was calculated and presented as the mean ± SEM. (**B**) MiR-29b target prediction. Left panel: The target regions of miR-29s on the 3′UTRs of MYCN. The target regions also exist in the recombinant luciferase mRNA transcribed from the wild-type plasmid MYCN-WT, but the 7 nucleotides of these regions were deleted in the transcripts of the mutant plasmid MYCN-MT. Right panel: Dual luciferase assay results. U87 cells were co-transfected with the recombinant luciferase plasmids. Luciferase activity of control group was normalized to 100%. (**C**) Correlation of miR-29b level and MYCN mRNA level in glioma tissues. (**D**) MYCN mRNA expression level in miR-29b over-expressed cells, detected by real-time PCR. GAPDH was used as internal control. (**E**) MYCN protein level in miR-29b over-expressed cells, detected by western blot assay. Results of control group were normalized to 100%. Data were presented as the mean ± S.E.M. (*n* = 4) Data were based on at least three independent experiments. *P < 0.05 vs. control group.

In order to study the functional roles of miR-29b in glioma, we chose two glioma cell lines, U87, U251, (Figure 1D) with the relative low expression levels of miR-29b to construct miR-29b over-expressed cell model for the functional study of miR-29b. (Figure 2A) The cell proliferation rates were determined by cell counting kit (CCK8) assay and 5-Ethynyl-2′- deoxyuridine (EdU) assay. As the results showed, the cell growth was significantly inhibited in cells treated with miR-29b transfectants. (Figure 2B–2F) The cell apoptosis levels were detected by flow cytometry. We observed that the in early-phase apoptotic rates were significantly increased in cells transfected with Lv-miR-29b in comparison with untreated or scramble treated control cells. (Figure 3A) Accordingly, we speculated that miR-29b could inhibit the proliferation and promote the apoptosis of glioma cells.

### MYCN is a direct target of miR-29b in glioma cells

To further investigate the molecular mechanisms of miR-29b in regulating the proliferation and apoptosis in glioma cells, we accessed the commonly cited database and performed the putative targets prediction for miR-29b using miRanda, TargetScan and PicTar. Consequently, we found that MYCN, a gene that has been reported as a significant regulator in the development and progression of glioma, was a candidate target of miR-29b. (Figure 3B) We also found that miR-29b share the same seed complementarity to MYCN 3′ UTR. Then, luciferase reporters assay created as pGL3-MYCN-WT-3′UTR and pGL3 -MYCN-MT -3′UTR revealed that miR-29b directly regulated MYCN expression via binding 3′UTR of MYCN in glioma cells. (Figure 3B) Moreover, we detected the expression of MYCN in normal brain tissues and glioma tissues. As a result, the expression of MYCN was significantly over-expressed with the ascending order of glioma grade, accompanying the down-regulation of miR-29b. (Figure 3C) Further, we also found that the expression of MYCN was significantly decreased in cells transfected with Lv-miR-29b. (Figure 3D and 3E).

### miR-29b inhibits the proliferation of glioma cells via MYCN dependent way

**Figure 4:**
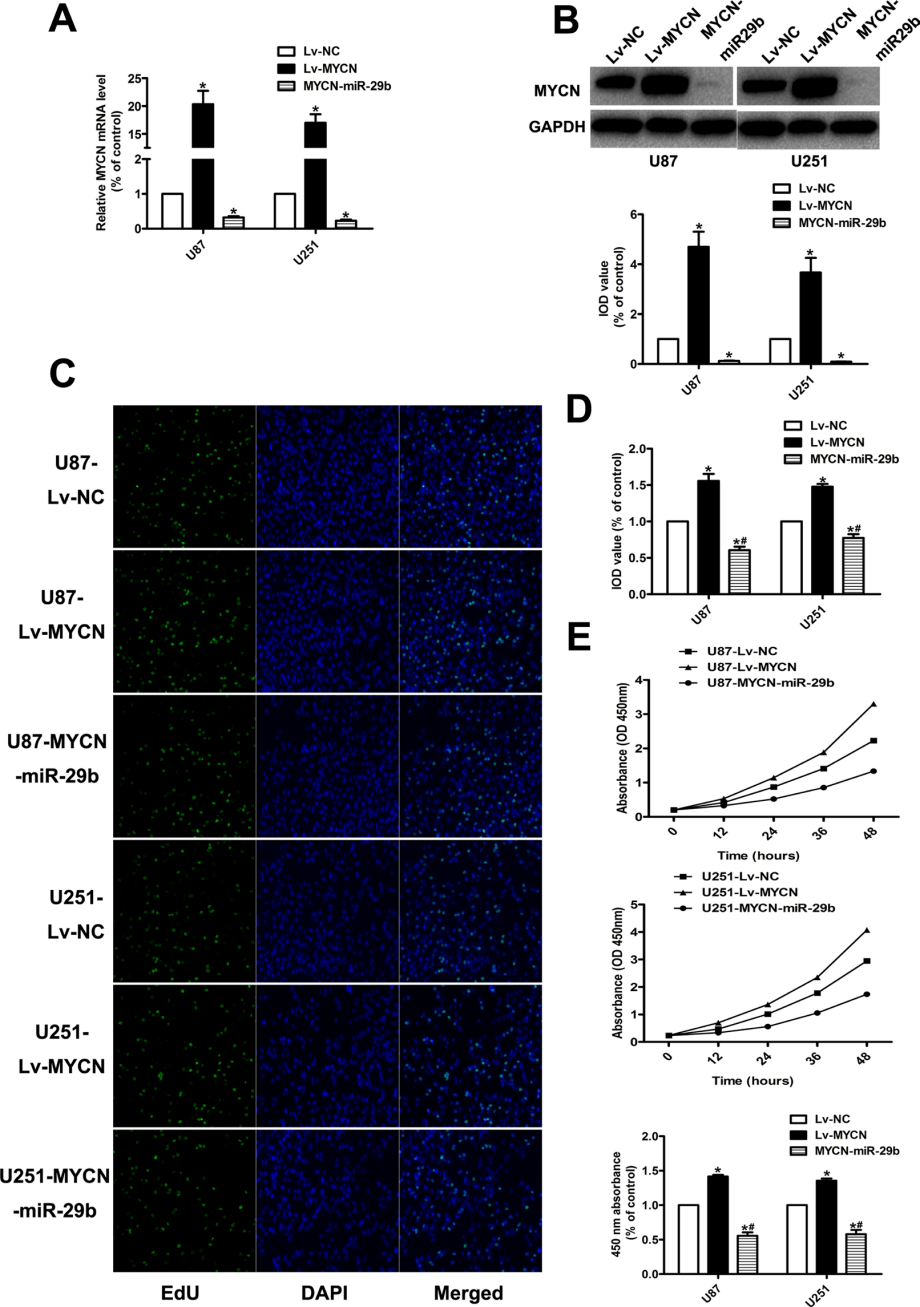
Effect of MYCN over-expression and co-transfection of Lv-MYCN and Lv-miR-29b on cell proliferation (**A**) Validation results of real-time PCR for MYCN over-expression, and co-transfection of Lv-MYCN and Lv-miR-29b (MYCN-miR-29b). GAPDH was used as internal control. (**B**) MYCN protein level in MYCN over-expressing cells and cells co-transfected with Lv-MYCN and Lv-miR-29b (MYCN-miR-29b), detected by western blot assay. (**C**, **D**) EdU results indicated over-expression of MYCN promoted cell proliferation, which was reversed by Lv-MYCN and Lv-miR-29b co-transfection (MYCN-miR-29b). Data were presented as the mean ± S.E.M. (**E**) CCK8 results demonstrated MYCN over-expression promoted cell proliferation, which was reversed by Lv-MYCN and Lv-miR-29b co-transfection (MYCN-miR-29b). Absorbance at 450 nm was presented as the mean ± SEM. Absorbance at 450 nm of cells treated with control plasmid was normalized to 100%. Data were collected and provided at 24 h after cultivation. (*n* = 4) Data were based on at least three independent experiments. *P < 0.05 vs. control group; ^#^*P* < 0.05 vs. Lv-MYCN group.

**Figure 5:**
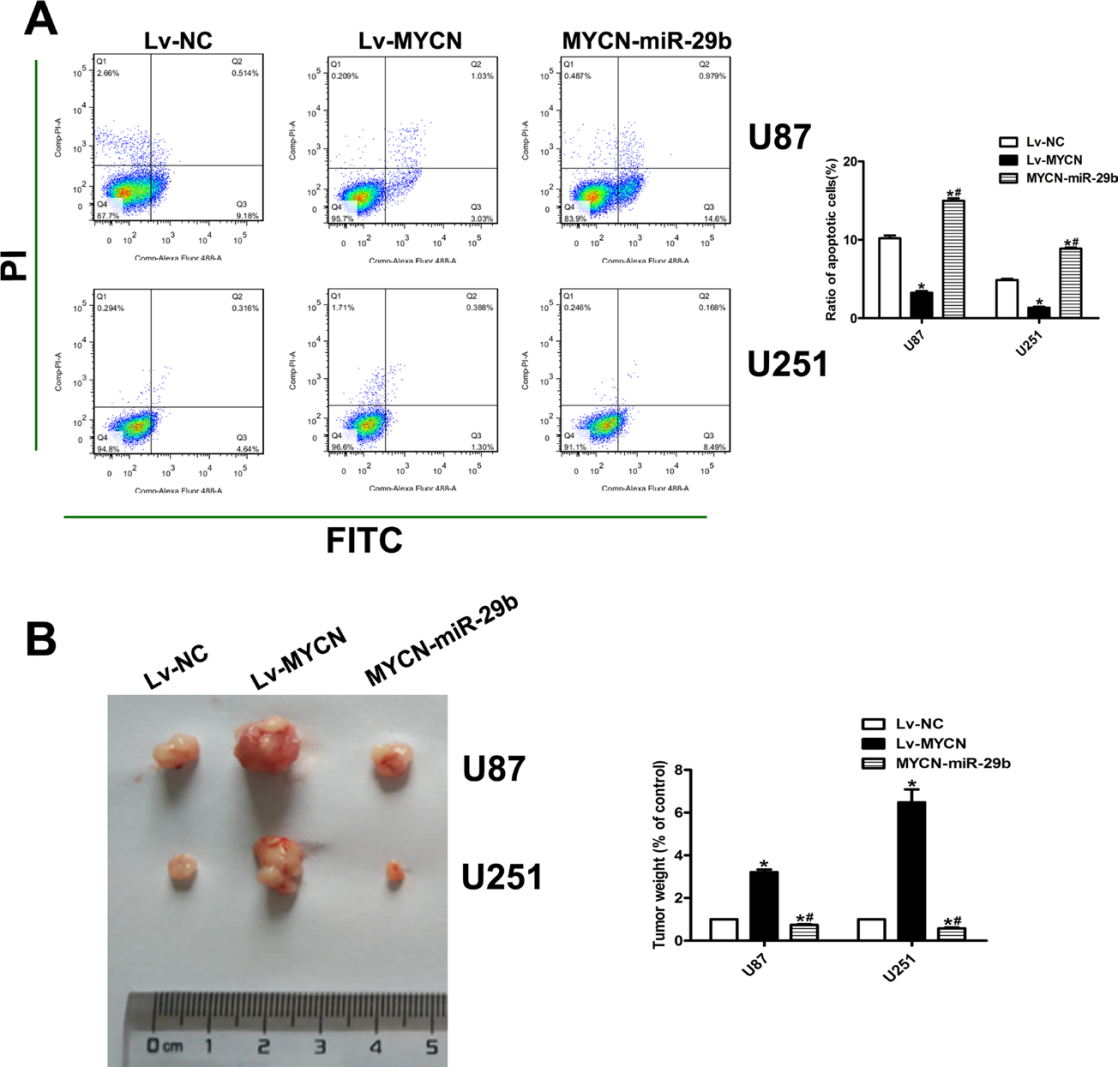
Effect of MYCN over-expression and co-transfection of Lv-MYCN and Lv-miR-29b on cell apoptosis and *in vivo* tumor growth (**A**) Cell apoptosis was assayed by FCA. Left panel: PI for cell nucleus staining; FITC for cytomembrane staining. Right panel: Ratio of early-phase apoptotic cells was calculated and presented as the mean ± SEM. (*n* = 4) (**B**) Left panel: Representative established tumor extracted from mice model established by subcutaneously inoculating with xenografts of glioma cells. (*n* = 6) Right panel: Tumor weight was calculated. The weight of control groups were normalized to 100%. Data were presented as the mean ± SEM. Data were based on at least three independent experiments. *P < 0.05 vs. control group; ^#^*P* < 0.05 vs. Lv-MYCN group.

To further demonstrate whether miR-29b regulated the proliferation and apoptosis through MYCN dependent way, MYCN were stably over-expressed in U87 and U251 cells combined with Lv-miR-29b transfection or not. (Figure 4A and 4B) As it showed, CCK8 and EdU indicated that the cell proliferation ability and anti-apoptosis ability was similarly significantly increased after over expression of MYCN; however, Lv-miR-29b transfection in MYCN over-expressing cells finally reversed the effect. (Figure 4C–4E and Figure 5A) The tumor-bearing mice model established by subcutaneously inoculating with xenografts of glioma cells presented consistent results. (Figure 5B).

### MiR-29b can be a biomarker for the diagnosis of glioma

**Figure 6:**
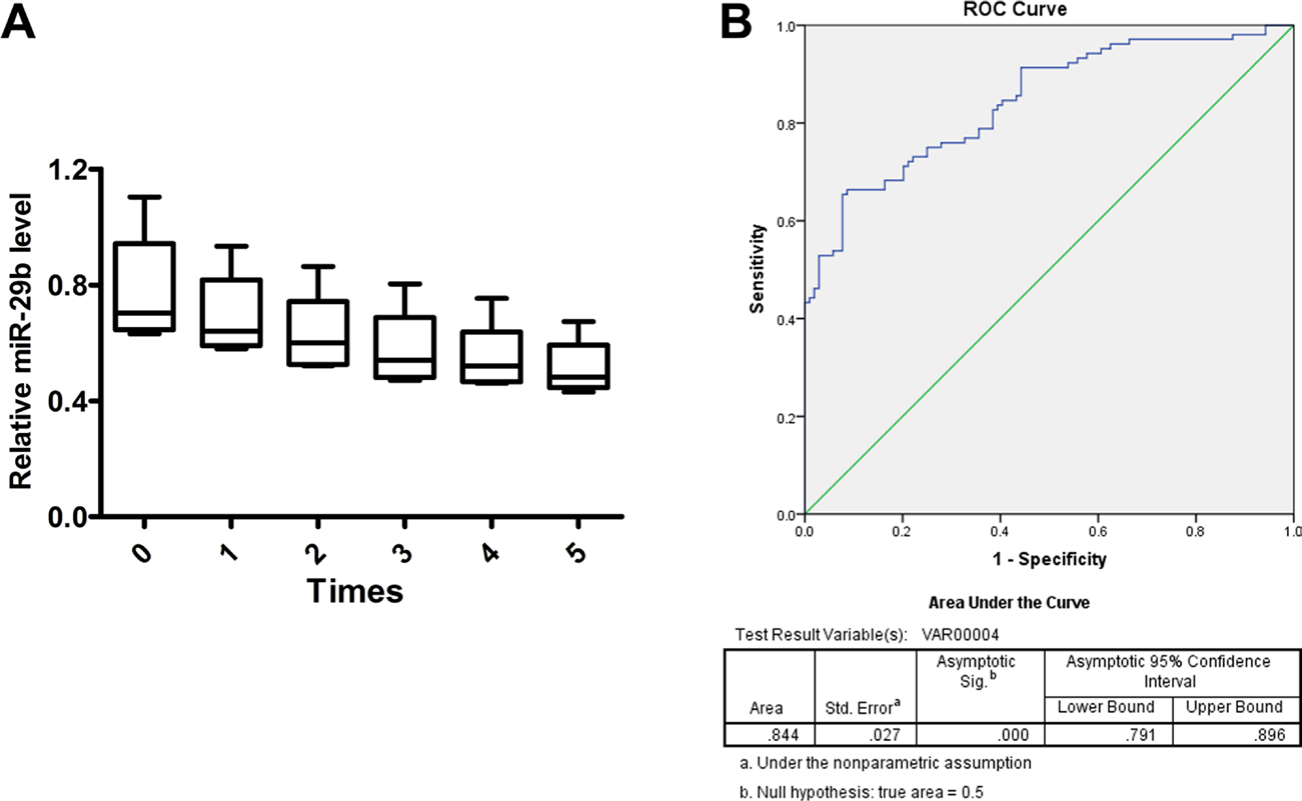
miR-29b as a biomarker for glioma diagnosis (**A**) Serum samples were frozen and thawed for five times, and expression stability of miR-29b in serum was detected by real-time PCR. Data were presented as box plots. Box plot explanation: upper horizontal line of box, 75th percentile; lower horizontal line of box, 25th percentile; horizontal bar within box, median; upper horizontal bar outside box, 95th percentile; lower horizontal bar outside box, 5th percentile. Data were based on at least three independent experiments. (**B**) Expression of miR-29b was detected in patients from whom plasma was obtained preoperatively, by comparison with healthy control. ROC curve analysis of miR-29b was used to detect the diagnostic efficiency of glioma. All experiments were performed in triplicate.

Previous studies have reported microRNAs as biomarkers for diagnosis and predictors for prognosis of glioma. Thus, to identify the effectiveness of miR-29b for glioma prediction, glioma serum samples from patients were collected before surgical resection and control serum samples (*n* = 104) were collected from individuals undergoing physical examination. Five serum samples were challenged with five-time frozen-thawed cycles to test the stability of miR-29b expression in serum; subsequently, expression level of miR-29b was detected by real-time PCR in all samples, which indicated that miR-29b was stably expressed in serum and may act as an effective predictor for glioma diagnosis (area under the curve, 0.844; *P* < 0.001). (Figure 6A and 6B).

## DISCUSSION

The past decade has witnessed the evolving progress of biological molecular therapies for diverse tumors and multiple biomarkers and biological targets have been found, several of which have been successfully applied in clinical tumor treatment, such as trastuzumab which is an antibody of human epidermal growth factor receptor type 2 (HER2) and nivolumab which is an antibody of the programmed cell death-1 (PD-1) [12–16]. Studies have also validated nonconding RNAs, which were formerly classified as RNA junk, as vital biomarkers and therapeutic biological targets for diverse human diseases, such as inflammatory diseases, metabolic diseases and cancers. And miRNAs, containing no more than 22 nucleotides, are the most studied nonconding RNAs [17–22].

In human tissues, miRNAs may regulate more than one-third of all human genes via binding loosely complimentary sequences within the 3′-untranslated regions (3′UTRs) of mRNA transcripts. Previous studies have demonstrated several miRNAs as important participators in glioma, such as miR-182 [23], miR-185 [24], which were abnormally expressed in plasma, therefore can be used as biomarker for the early diagnosis of glioma, and miR-21 [25], miR-125b [26] and miR-205 [27] which were deregulated in glioma tissues, thus can be potential therapeutic targets for glioma. In this study, we focus on a miRNA, termed miR-29b, which was down-regulated in glioma.

Previous studies have suggested the abnormal existence of miR-29b in glioma and predicted its potential role in the genesis of glioma; however, no validation with large number of clinical samples was performed. Here, in order to further through light on the actual functional participation of miR-29b in glioma, we carried out the present study. We found that miR-29b was down-regulated in glioma tissues, and more significant differences were observed in higher level gliomas. The evaluation of miR-29b in serum samples indicated the similar results which were consistent with those in tissue samples. The correlation analysis further demonstrated that the expression levels miR-29b showed significant relevance in glioma tissues and in corresponding plasma samples. Then we studied the function of miR-29b in regulating the cell behavior. We found that in miR-29b over-expressed U87 and U251 cells, cell proliferation and anti-apoptosis ability were significantly inhibited. We additionally predicted the targets of miR-29b with miRanda, TargetScan and PicTar, and finally we found MYCN, a previously reported tumor promoter in glioma carcinogenesis [28, 29], shared the same seed complementarity to MYCN 3′ UTR, which was further validated by luciferase report assay. We also detected the expression of MYCN in glioma tissues, and found that the expression of miR-29b was negatively correlated with the mRNA and protein levels of MYCN. Then we assayed the expression of MYCN in miR-29b over-expressed cells and found that MYCN can be down-regulated by miR-29b. In MYCN over-expressing cells, cell proliferation and anti-apoptosis ability were promoted; however, these effects can be reversed by miR-29b over-expression. Lastly, we evaluated the effectiveness of miR-29b for glioma prediction, which indicated that miR-29b was stably expressed in serum and can be an effective predictor for glioma diagnosis.

In the present study, we for the first time verified the abnormally down regulation of miR-29b in glioma with large number of clinical samples, and we found miR-29b may act as a tumor inhibitor via MYCN dependent pathway. We also demonstrated miR-29b as a serum biomarker for glioma diagnosis. Our study combining with previous findings provided miR-29b as a promising therapeutic target and diagnostic biomarker for glioma. Nonetheless, further studies with larger scale of samples and studies focusing on the precise mechanism of the interaction between miR-29b and MYCN are warranted.

## MATERIALS AND METHODS

### Patients and clinical samples

Fresh glioma specimens, paired peripheral blood samples and normal brain tissue samples were obtained from patients undergoing surgery in Yancheng First People's Hospital (Yancheng, China) between 2006 and 2011. The histopathologic diagnoses were performed by the pathologists according to WHO criteria. Peripheral blood samples from healthy persons were randomly selected at Yancheng First People's Hospital. The study was approved by our Institutional Ethics Committee. Our research was performed according to the government policies and Helsinki Declaration. All participants signed the written informed consent. Peripheral blood samples were collected before surgical operation. Normal brain tissues were collected from the temporal lobes and saddle area of the patients with arachnoid cyst (AC) after surgery. Clinical and pathological information are summarized in Table 1.

**Table 1:** The clinicopathological relevance analysis of miR-29b expression in glioma patients

Feather	All patients	miR-29b		*P*-value
		Low expression (< median)	High expression (≥ median)	
All cases	104	52	52	
Age, years				**< 0.001**
< 50	37	10	27	
> = 50	67	42	25	
Gender				0.665
Male	74	38	36	
Female	30	14	16	
WHO grade				**< 0.001**
I	14	6	8	
II	48	15	33	
III	26	17	9	
IV	16	14	2	
KPS				**0.002**
< 90	68	48	20	
> = 90	36	14	22	

Total data from 104 glioma patients were analyzed. For the expression of miR-29b, median expression level was used as the cutoff. Data were analyzed by chi-squared test. *P*-value in bold indicates statistically significant.

### Cell lines and cell transfection

Human glioma cell lines, U87, LN229, H4 and U251, were purchased from the Shanghai Institute of Biochemistry and Cell Biology (Chinese Academy of Sciences, Shanghai, China). All cell lines were cultured routinely in Dulbecco's modified Eagle's medium (DMEM) (Invitrogen, Grand Island, NY, USA) added with 10% heat-inactivated fetal bovine serum (FBS) (Gibco, Carlsbad, CA, USA), L-glutamine (2 mM), penicillin (100 U/ml), and streptomycin (100 mg/ml) and were maintained in 5% CO2 at 37°C. The synthesised and purified miRNA or MYCN gene fragment was inserted into a lentivirus vector (pll3.7), named Lv-miR-29b or Lv-MYCN, cells co-transfected with Lv-MYCN and Lv-miR-29b were termed as MYCN-miR-29b groups. Cells were transfected with the packaged recombinant lentivirus combined with polybrene (8 μg/ml) to construct MYCN or miR-29b stably over-expressed cell model. Parallel control group (Lv-NC) was constructed using the lentivirus plasmid and empty vector plasmid. After 48 h transfection, cells were collected or harvested.

### Real-time polymerase chain reaction (PCR)

TRIzol reagent (Invitrogen, CA, USA) was used to extract the total RNAs from tissues and cells. NanoDrop 2000/2000c (Thermo Scientic, MA, USA) was used to check the quantity and integrity of the isolated RNA. Reverse transcriptase kit (TaKaRa, Dalian, China) was used to get cDNA from RNA (500 ng). Real-time PCR was performed using ABI Prism 7900HT (Applied Biosystems, Foster City, CA, USA) according to the direction of the reagents and results were analyzed in triplicate assays. MYCN primer sequence: forward (5′∼3′) GTCACCACATTCACCATCAC, MYCN reverse (5′∼3′) GGGAAGGCATCGTTTGAG; GAPDH primer sequence: forward (5′∼3′): GCCTCGTCCCGTAGACAAAA; reverse (5′∼3′) GATGGGCTTCCCGTTGATGA. U6 and GAPDH mRNA were used as internal controls.

### Western blotting

Proteins extracted from cells and fresh tissues were quantified with the Bradford assay kit (Bio-Rad Laboratories, Hercules, CA). Samples were lysed using radio-immunoprecipitation assay buffer added with fresh protease and phosphatase inhibitors (Beyotime, Nantong, China). Protein samples of equal amounts (30 μg) were loaded to each lane, separated by SDS-PAGE and then transferred to a polyvinylidene fluoride membrane. Antibodies against MYCN (CST, MA, USA) and GAPDH (CST, MA, USA) were used in immunoblotting as described previously [30]. Immunoreactive bands were visualized using the ECL kit (Pierce, Rockford, IL, USA) and integrated density of the bands was quantified by ImageJ software (NIH, Bethesda, MD, USA)

### Animals and subcutaneous xenotransplantation model

BALB/c nu/nu male mice (4–6 weeks old) were purchased from the Laboratory Animal Center of Yangzhou University. All animals were maintained under specific pathogen-free conditions and received humane care. All experiments were carried out are according to the guidelines outlined in the Guide for the Care and Use of Laboratory Animals. Cells (5×10^6^) with stable over-expression of MYCN combined with miR-29b transfection or not in U87 and U251, and control cells (Lv-NC) were subcutaneously implanted into the bilateral axillae of the BALB/C nude mice. Four weeks later, all mice were sacrificed. Tumor tissues were integrally stripped out and the weight of each tumor was measured.

### Flow cytometry analysis (FCA)

Cells were treated with 0.08 mM H2O2 for 2 h for stimulation of apoptosis, and then cell apoptosis detection was performed with an Annexin V-FITC/PI Apoptosis Detection Kit (Vazyme Biotech, China). Cells prepared were analyzed on a FACS Calibur flow cytometer equipped with CellQuest software (BD Biosciences, New York, NY, USA).

### Cell proliferation assay

A CCK8 kit (Vazyme Biotech, China) and a Cell-Light EdU Apollo567 *In Vitro* Kit (RiboBio, Guangzhou, China) were used for the evaluation of cell proliferation. For CCK8 assay, cells (2 × 10^3^) were seeded into 96-well plates, cultured for 12, 24, 36 and 48 hours, and CCK8 reagent was then added to each well and incubated for 2 h at 37°C. The measurement of absorption was performed using microplate reader at 450 nm (ELX-800; Bio-Tek, Winooski, VT, USA). For 5-ethynyl-2′-deoxyuridine (EdU) assay, cells (2 × 10^5^) were seeded into Glass Botttom Cell Culture Dishes (Nest Biotechnology, NJ, USA), then cells were treated according to the given instruction. Prepared samples were detected with a laser confocal scanning microscopy.

### Statistical analysis

Data were analyzed with GraphPad (GraphPad Software, San Diego, CA, USA) and SPSS software (SPSS Inc., Chicago, IL, USA) and showed as mean ± SEM. Student's *t* test and χ^2^ test were used to evaluate statistical differences in different groups. Two-tailed tests were applied to all data if not specified. One-way ANOVA analysis followed by LSD test was performed for multiple comparisons. The effectiveness of miR-29b for glioma prediction was performed with receiver operating characteristic curve (ROC) analysis using SPSS software (SPSS Inc., Chicago, IL, USA). All experiments were performed for triple times. *P* < 0.05 was considered statistically significant.
